# The Role of 3-*epi*-18*β*-Glycyrrhetinic Acid and Hepatic Function in Licorice-Induced Pseudohyperaldosteronism

**DOI:** 10.1016/j.ekir.2026.103771

**Published:** 2026-01-09

**Authors:** Tetsuhiro Yoshino, Ryota Sakoda, Toshiaki Makino

**Affiliations:** 1 Center for Kampo Medicine, Keio University School of Medicine, Tokyo, Japan; 2 Department of Pharmacy, Kochi Medical School Hospital, Kochi, Japan; 3 Department of Pharmacognosy, Graduate School of Pharmaceutical Sciences, Nagoya City University, Nagoya, Japan

To the Editor:

We read with great interest the case series by Bendjilali-Sabiani *et al.*^1^ regarding licorice-induced pseudohyperaldosteronism. The authors reported a remarkably prolonged elimination half-life of glycyrrhetinic acid (GTA; also referred to as GA) in a patient with alcoholic cirrhosis (128.1 hours in case 1), compared with the theoretical 10 to 30 hours. This finding provides crucial pharmacokinetic evidence supporting our previous retrospective cohort study, which identified liver dysfunction as a significant risk factor for pseudoaldosteronism,^2^ reflecting the critical role of hepatic metabolic and transporting systems in its clearance.

Although the authors cited 3-monoglucuronyl-GTA as a causative agent, we offer a pharmacokinetic refinement. Although 3-monoglucuronyl-GTA is produced in the intestine, our investigations using specific analytical methods demonstrated that it is rarely detected in human circulation.^3^ Thus, the authors' decision to measure plasma GTA—rather than 3-monoglucuronyl-GTA—was methodologically appropriate.

However, the gut microbiota’s role extends beyond hydrolysis. We recently demonstrated that gut bacteria also facilitate GTA isomerization, producing 3-*epi*-18*β*-GTA.^4^ We detected this C-3 epimer in human serum and established its correlation with the risk of pseudoaldosteronism.^4^ Furthermore, our latest toxicokinetic investigation revealed that 3-*epi*-18*β*-GTA exhibits significantly delayed elimination compared with GTA because of delayed hepatic metabolism and biliary clearance.^5^

Because the specific detection method was not detailed in the authors’ report, the “GTA” measured may represent a mixture of GTA (18*β*-GA) and 3-*epi*-18*β*-GTA. The extreme half-life prolongation in their hepatically impaired patient is consistent with the accumulation of this poorly excreted epimer. Recognizing 3-*epi*-18*β*-GTA is essential for understanding individual susceptibility.
